# Errors in aerial survey count data: Identifying pitfalls and solutions

**DOI:** 10.1002/ece3.8733

**Published:** 2022-03-18

**Authors:** Kayla L. Davis, Emily D. Silverman, Allison L. Sussman, R. Randy Wilson, Elise F. Zipkin

**Affiliations:** ^1^ 3078 Department of Integrative Biology Michigan State University East Lansing Michigan USA; ^2^ 3078 Ecology, Evolution, and Behavior Program Michigan State University East Lansing Michigan USA; ^3^ Migratory Bird Program U.S. Fish and Wildlife Service Laurel Maryland USA; ^4^ U.S. Geological Survey Eastern Ecological Science Center at the Patuxent Research Refuge Laurel Maryland USA; ^5^ Migratory Bird Program U.S. Fish and Wildlife Service Jackson Mississippi USA

**Keywords:** abundance, aerial survey, count data, counting error, imperfect detection, nondetection, species misidentification, study design

## Abstract

Accurate estimates of animal abundance are essential for guiding effective management, and poor survey data can produce misleading inferences. Aerial surveys are an efficient survey platform, capable of collecting wildlife data across large spatial extents in short timeframes. However, these surveys can yield unreliable data if not carefully executed. Despite a long history of aerial survey use in ecological research, problems common to aerial surveys have not yet been adequately resolved. Through an extensive review of the aerial survey literature over the last 50 years, we evaluated how common problems encountered in the data (including nondetection, counting error, and species misidentification) can manifest, the potential difficulties conferred, and the history of how these challenges have been addressed. Additionally, we used a double‐observer case study focused on waterbird data collected via aerial surveys and an online group (flock) counting quiz to explore the potential extent of each challenge and possible resolutions. We found that nearly three quarters of the aerial survey methodology literature focused on accounting for nondetection errors, while issues of counting error and misidentification were less commonly addressed. Through our case study, we demonstrated how these challenges can prove problematic by detailing the extent and magnitude of potential errors. Using our online quiz, we showed that aerial observers typically undercount group size and that the magnitude of counting errors increases with group size. Our results illustrate how each issue can act to bias inferences, highlighting the importance of considering individual methods for mitigating potential problems separately during survey design and analysis. We synthesized the information gained from our analyses to evaluate strategies for overcoming the challenges of using aerial survey data to estimate wildlife abundance, such as digital data collection methods, pooling species records by family, and ordinal modeling using binned data. Recognizing conditions that can lead to data collection errors and having reasonable solutions for addressing errors can allow researchers to allocate resources effectively to mitigate the most significant challenges for obtaining reliable aerial survey data.

## Graphical abstract

We highlight common challenges associated with collecting and analyzing aerial survey data to estimate abundance including nondetection, counting error, and species misidentification. Using a literature review, case study, and an online quiz, we explore the pros and cons of available solutions to overcome each issue. We conclude by providing specific suggestions for practitioners designing aerial surveys. This paper provides clarity on the possible errors that can be introduced in aerial survey data, guides researchers via reasonable approaches to address ongoing issues, and identifies areas for future research.

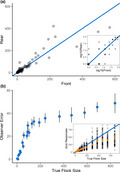

## 1 INTRODUCTION

Reliable estimates of wildlife abundance are imperative for understanding how environmental variables influence population and community dynamics, assessing trends across time and space, and guiding conservation and management decisions (Williams et al., 2002). Most estimates of wildlife abundance are derived from surveys that collect count data on target species (Elphick, 2008). These surveys are typically designed to yield counts of the species within predefined sampling units for a fixed amount of sampling effort (e.g., observation time, travel speed) to make inferences on abundance across a study region. Differing sampling designs, methods, and analysis techniques for count‐based surveys can vary in their ability to yield accurate and precise estimates of abundance. Poorly conducted surveys can produce data that obscure animal–environment relationships or introduce biases into inferences (Conroy et al., 2008).

For species that occur at low densities or across large spatial areas, aerial surveys are often the most efficient platform to collect observational count data (Caughley, 1977; Parker et al., 2010). Aerial surveys typically consist of flight transects in which observers count individuals of the target species along a transect line or strip or within the boundaries of a sampling plot from a fixed‐wing aircraft or helicopter (Caughley, 1977; Jolly, 1969). Aerial surveys have a long history in ecological research, starting with censuses of North American ungulate populations in rugged and remote terrain in the 1940s (Buechner et al., 1951; Hunter & Yeager, 1949). Researchers have long recognized the distinct advantages of aerial surveys, including the ability to rapidly collect data across large spatial extents (Keeping et al., 2018; Lee & Bond, 2016). Comparable ground‐based surveys can take weeks to cover the same area that an aerial survey can cover in a matter of days (Keeping et al., 2018). As such, systematic aerial survey designs can be more cost‐effective than ground‐based surveys despite aerial surveys being considerably more expensive per unit time (Keeping et al., 2018; Khaemba et al., 2001). In remote environments or rugged terrain, wildlife monitoring is often only feasible with aerial surveys. Ground‐based terrestrial surveys are typically limited to areas with road systems or areas that can be safely traversed by foot (Jachmann, 1991), while vessel‐based surveys of marine and aquatic environments are slower‐paced and best suited for remote regions far from land (Briggs et al., 1985). In many situations, aerial surveys are the preferred—and sometimes only—method for data collection on wide‐ranging species, including those that occupy remote environments (Conn et al., 2013), are highly mobile, or are difficult to count from the ground because of body size, coloration, or cryptic behaviors (Greene et al., 2017). In addition, data from aerial surveys may be summarized into a meaningful index of abundance for tracking changes in species' populations and distributions over time (Amundson et al., 2019; Chirima et al., 2012; Finch et al., 2021; Obbard et al., 2018). However, such indices are subject to biases, particularly if surveys are not standardized and/or errors in data collection are not constant through time.

While aerial surveys offer many benefits, the method also presents challenges for high quality inferences on species abundance. Mistakes resulting from imperfect observer detection during sampling can introduce errors into the data. As with other survey types, common manifestations of imperfect detection in aerial surveys include: nondetection (failure to detect an individual or group even though it is present), counting error (inaccurate enumeration of group size), and species misidentification (incorrectly identifying the species of an individual). Nondetection errors occur because an individual that is available to be seen is missed or because an individual is unavailable for detection (e.g., temporarily outside of the survey unit, under vegetation or water and not exposed to sampling; Kéry & Schmidt, 2008). For example, ungulates, such as mule deer (*Odocoileus hemionus*), can be difficult to detect with aerial surveys in certain cover types and vegetation density (Zabransky et al., 2016), which can result in a failure to record all individuals on a survey transect. Counting errors can result in observers either over‐ or under‐recording the true number of individuals on a transect. Counting errors may also occur as a product of species behavior or the survey platform itself (e.g., fixed wing versus helicopter surveys). Many species, including mid‐sized marine mammals, aggregate in large numbers and are highly mobile, making it difficult to accurately enumerate group sizes from fast moving aircraft (Gerrodette et al., 2019). Counting errors are often treated as a failure to detect to individuals (i.e., as a nondetection) and common methods for estimating nondetection (e.g., through detection probabilities) can address minor counting error issues. However, such methods cannot accommodate severe counting errors, such as those that might occur when large groups are encountered. Species misidentification can be a bi‐directional issue if a survey focuses on multiple species, resulting in an over‐count of one species and an undercount of another. Although observers may be able to detect small‐bodied animals, such as many waterbird species, they may be difficult, or nearly impossible, to correctly identify (Johnston et al., 2015) due to the speed of the aircraft and distance from the observer.

In this paper, we provide an overview of the current challenges to estimating species’ abundance using aerial survey data. We reviewed the literature on aerial wildlife survey methods over the last 50 years to examine how each major issue manifests across species and environments. Several challenges of using aerial survey data have not been adequately discussed in the literature despite their persistence in aerial survey data, likely because in many cases, there is no obvious approach to adequately address these issues. Often, issues such as counting error and misidentification are ignored during analyses of count data, either because researchers do not recognize they are present, cannot estimate the magnitude of errors, or they are unable to account for the errors directly during analysis (Clement et al., 2017). Thus, in addition to our literature review, we highlight how aerial survey challenges can manifest using a case study of waterbird aerial surveys in the Gulf of Mexico and an online quiz of aerial observers. The case study data come from an aerial survey that implemented a double‐observer method and are therefore ideal to investigate both how errors can arise in aerial count data and also how they might be addressed through data analysis. Additionally, issues of misidentification and counting error are prevalent in waterbird data due to frequent aggregations of multispecies groups. The online quiz further highlights this issue of counting error, particularly how the magnitude of observer counting error changes as group size increases. The purpose of our review is to provide clarity on the possible errors that can be introduced in aerial survey data but are often ignored, guide researchers to reasonable approaches to ameliorate ongoing issues, and identify areas for future research.

## 2 METHODS

### 2.1 Literature review

We searched Web of Science and Google Scholar using the key words: “aerial survey*,” “aerial wildlife survey*,” “aerial survey issue*,” “aerial survey error*,” and “aerial survey method*.” We limited our search to peer‐reviewed articles published between 1970 and 2020. This cutoff ensured a large sample size through time while also excluding the earliest papers describing methods and technologies that are no longer relevant. Our inclusion criteria required that the article: (1) contain aerial survey methodology for in‐flight observer surveys, and (2) discuss the implications of the methodology on the accuracy or precision of count data on subsequent inferences. We did not include papers that only reported results from aerial survey work. In most cases, included papers were methods‐focused, generally on specific aspects of aerial survey design and implementation. We inspected the titles and abstracts of all articles of the first fifty results returned by each key word (*n* = 5) from the two search engines and discarded articles that did not fit the inclusion criteria. We read all papers that passed this initial inspection and further refined our collection based on the inclusion criteria. We also searched the literature cited of all articles that passed our inspection to ensure we did not overlook any critical literature. For each of the articles that met the inclusion criteria, we identified the major issues encountered and categorized the type of issue. We also identified the system and taxa in which the study was conducted, aircraft type, and the sampling style or design (line transect, strip transect, systematic sampling, double observer, mark‐recapture, distance sampling; Appendix S1). Because our review is a synthesis of the relevant literature instead of a systematic review, all quantitative metrics from our literature review reported herein should be considered indicative of general trends within aerial survey literature.

Issues of imperfect detection are conflated in the literature because most models used to estimate abundance are unable to simultaneously parse multiple sources of observation error, such as unobserved individuals versus misidentified and miscounted individuals (but see Clement et al., 2017 for an interesting exception). For the purposes of this review, we distinguish nondetection, counting error, and species misidentification as distinct issues. Developing effective mitigation strategies for aerial survey methods requires understanding the different potential sources of error. Left uncorrected, these various detection errors may act differentially to bias count data.

### 2.2 Waterbirds case study

Our interest in aerial survey biases is motivated by planned analysis of data generated by the Gulf of Mexico Marine Assessment Program for Protected Species (GoMMAPPS). We used the GoMMAPPS data to examine the issues commonly encountered in aerial surveys and how challenges with data analysis can be addressed in practice.

**FIGURE 1:**
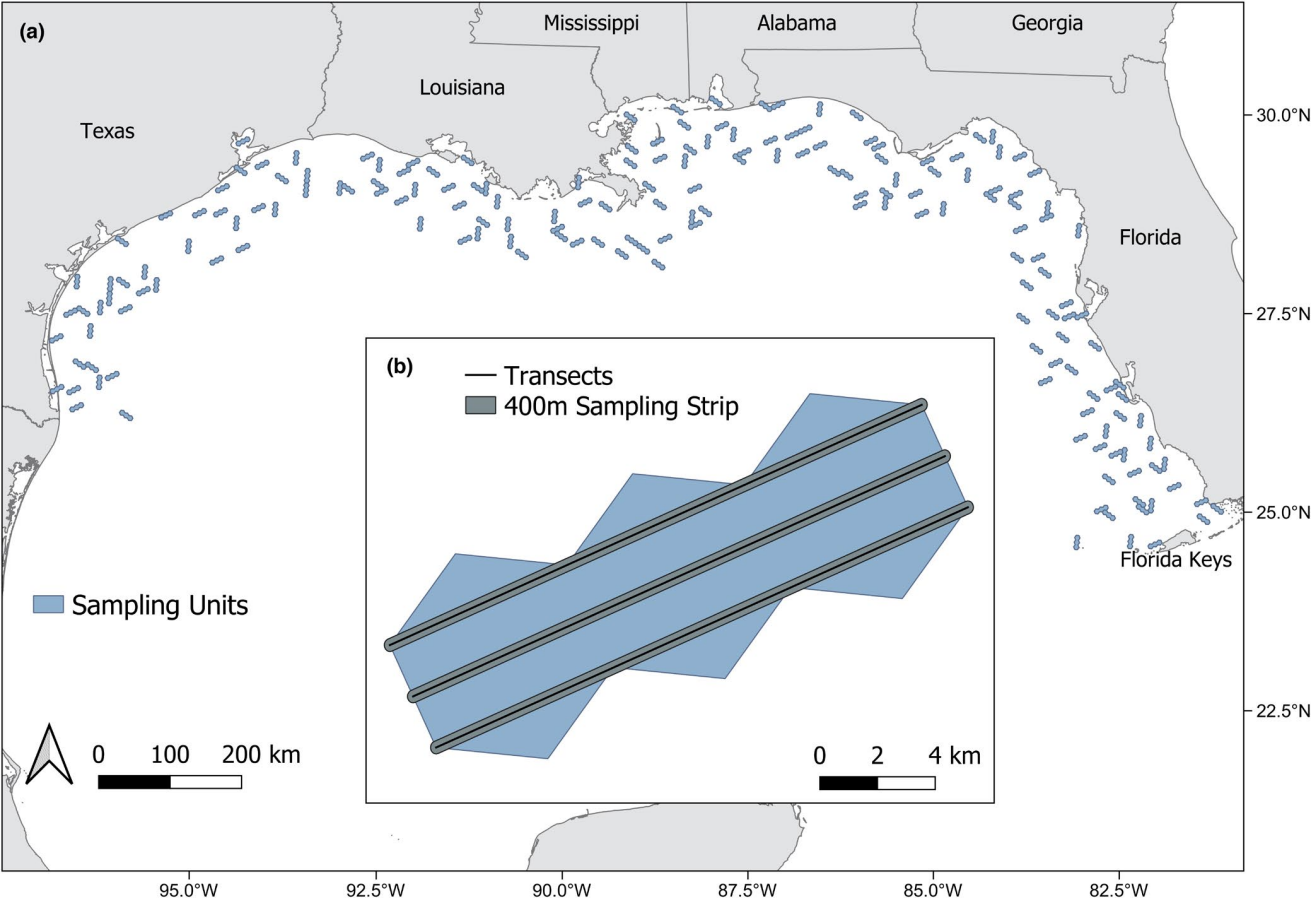
(a) Gulf of Mexico Marine Assessment Program for Protected Species survey units (*n* = 180) for summer and winter 2018–2020 surveys. (b) Schematic diagram depicting the design of a single survey unit (inset). Three transect lines (black lines) were placed inside the survey unit, and observers counted and identified all waterbirds within 400 m of the transect line (200 m on each side of the transect; shown in gray)

The GoMMAPPS project conducted aerial surveys across the northern Gulf of Mexico to identify and count all detected waterbirds in the nearshore environment during approximately two‐week long surveys in summer 2018 and winter 2018–2020. We randomly selected survey units (*n* = 180 of 5866 units) from the U.S. Environmental Protection Agency's Environmental Monitoring and Assessment Program (U.S. EPA EMAP) 40 sq. km hexagon grid dataset (White & U.S. EPA, 1992) using a generalized random tessellation stratified (GRTS) design (Stevens & Olsen, 2004) that covered the nearshore environment (coastline to 50 nm offshore) from the Texas‐Mexico border to the Florida Keys (Figure 1a). Surveys of each hexagon occurred along three transects that were parallel to each other, with each ~21 km spanning the length of the selected hexagonal survey unit and two neighboring units. We also randomized orientation (aircraft approach direction) of each of the chosen units. Observers surveyed the same 180 40‐km^2^ hexagonal units (or a subset of these due to weather constraints: winter 2019, *n* = 111 and winter 2020, *n* = 130) in each survey event (single survey season, e.g., winter 2018). During surveys, in‐flight observers counted and identified (to the lowest taxonomic level) all waterbirds within a 400 m strip transect (200 m on either side of the flight transect; Figure 1b). Surveys were flown at 110 knots and an altitude of 61 m, which precluded use of a distance‐sampling approach to estimate detection probabilities because detection does not decrease substantially across the width of the strip transect at this height (Certain & Bretagnolle, 2008). For our analyses, we focus on aerial waterbird surveys conducted in winter and summer 2018, winter 2019, and winter 2020.

To examine detection errors, data were collected with a double‐observer protocol where same‐side front‐ and rear‐seat observers independently recorded count and species identification records of all waterbirds that they observed in the observation strip (flight transect out to 200 m). Two experienced observers, a pilot‐biologist and a crew member, were always stationed in the front seats of the plane and counted out of their respective windows. A second experienced observer (another crew member) sat in a rear seat either behind the pilot‐biologist or behind the first crew member for the double‐observer protocol. The two crew members rotated their seat positions throughout the survey so crew member detection could be evaluated independently of seat position. Observers (pilot‐biologist and crew members) recorded the species (or taxonomic family when species identification was not possible), number of individuals in the group (one or more), and the GPS location. During post hoc data processing, we grouped double‐observer records that were recorded within 10 s of each other. We chose this temporal cutoff to accommodate differences in visibility between observers and potential lags in recording time. For example, front observers could see further ahead of the aircraft than the rear observers, and this visibility difference may have produced recording lags for the rear observer. Thus, the 10‐s window limited double‐observer records to those most likely to contain matching records. Grouped double‐observer records were then classified as: Species + Count Match – count and species identification matched between observer records, Generic + Count Match – count and taxonomic family matched between observer records, Species + Bin Match – log10 count bin (i.e., 0, 1–10, 11–100, 101–1000, and 1000+) and species identification matched between observer records (after count matches accounted for), Generic + Bin Match – log10 count bin (i.e., 0, 1–10, 11–100, 101–1000, and 1000+) and taxonomic family matched between observer records (after count matches accounted for), Species Only Match—species identification matched but neither count nor count bin matched between observer records, Generic Only Match—species taxonomic family matched but neither count nor count bin matched between observer records, Mismatch—species did not match between observer records, and No Match—there was no observation from the other observer recorded within 10 s. We note that the use of the term “generic” is meant in the generic sense to be interpreted as “general,” not in the taxonomic sense to be interpreted as “genera.” This double‐observer protocol and data processing procedure allowed us to identify potential errors, including nondetection, counting error, and misidentification.

### 2.3 Online quiz

We conducted an online survey to evaluate observer counting errors with known group‐size data. We designed the group (flock) counting quiz using Qualtrics survey software. The design and content of the quiz were adapted from the U.S. Fish and Wildlife Service Aerial Observer Training and Testing Resources (https://www.fws.gov/waterfowlsurveys/). We distributed the quiz via email to ~100 trained aerial observers (including those from the GoMMAPPS project) and biologists with no aerial survey observer experience. Seventy‐eight individuals completed the online quiz. The quiz consisted of background questions regarding respondents’ level of experience conducting aerial bird surveys (Expert, Intermediate, Novice, No Experience; Appendix S2, Table A1) and confidence in their flock counting skills (High, Medium, Low; Appendix S2, Table A2). The flock counting portion of the quiz consisted of two practice images and 22 timed quiz images of known‐size flocks (Appendix S2, Table A3 and Figure A1) that were representative of flock sizes observed during GoMMAPPS surveys (Appendix S2, Figure A2). Each image was displayed for 10 s before it disappeared, and the quiz automatically advanced to a question asking how many birds were in the image. See Appendix S2 for additional details.

## 3 RESULTS

### 3.1 Data summaries

#### 3.1.1 Literature review

**FIGURE 2:**
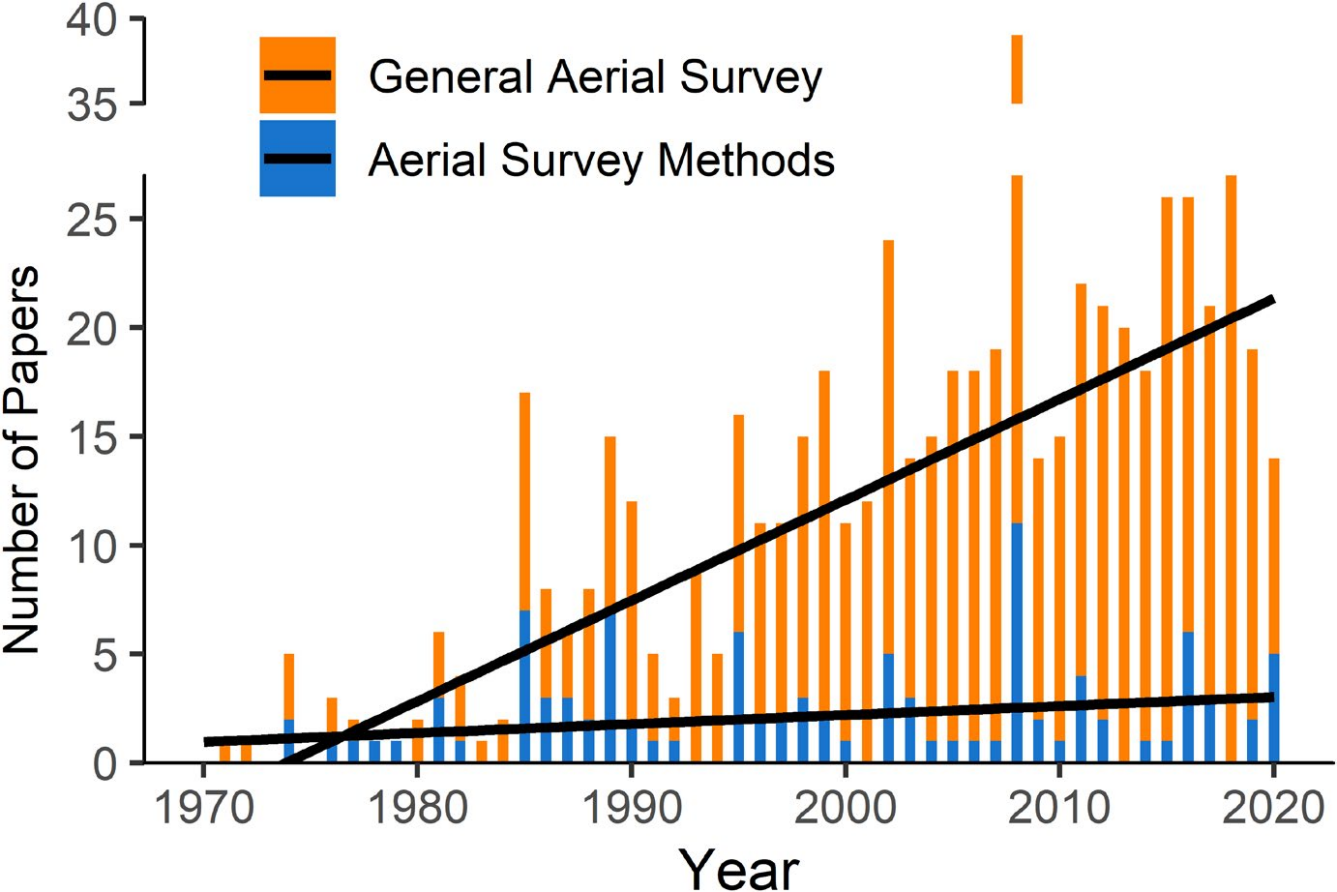
Aerial wildlife survey papers published by year during 1970–2020. The methods focused papers included in our review are shown in blue, and the general aerial survey literature are shown in orange. Black lines show regressions for the general aerial survey literature and aerial survey methods literature, respectively. The general aerial survey literature consists of the first 500 results of the following Google Scholar query: “aerial survey*” AND helicopter OR fixed‐wing OR aircraft OR plane AND count OR abundance AND wildlife OR ecology OR conservation

Of the 108 items returned by the Web of Science search and the 250 items returned by the Google Scholar search, 102 papers that were published from 1974 to 2020 met our inclusion criteria (Appendix S1). Although the number of peer‐reviewed publications using aerial survey methods has increased over time, papers focused specifically on methodology of aerial surveys have not exhibited the same trend (Figure 2). Most of the papers in our collection examined a single species (*N* = 49) or taxonomic group (*N* = 37), but six papers focused on multiple taxa, three were based on simulations, and seven did not name specific species of interest. The taxonomic groups studied were birds (*N* = 26), marine mammals (*N* = 11), terrestrial ungulates (*N* = 47), and macropods (*N* = 8). The aerial surveys covered a wide range of environments including terrestrial (i.e., savanna, forest, mountain; *N* = 64) and aquatic/marine (i.e., open‐ocean, nearshore, wetlands; *N* = 31) across North America (*N* = 47), Australia (*N* = 23), Africa (*N* = 16), and Europe (*N* = 5). Fixed‐wing aircraft were used most frequently (*N* = 43), but helicopters were also employed in a sizable portion of projects (*N* = 26). Seven papers used both types of aircrafts, and 26 either did not name the aircraft type or it was not applicable because the study used aerial photos or simulated data. Approximately one‐third (32.4%; *N* = 33) of papers discussed survey design variables including the size, shape, and configuration of survey units, delineation of survey units across a study area, and the timing and cost of surveys. The most common aerial survey designs included transect‐based designs, primarily strip transects (*N* = 61), quadrat (*N* = 8) or complete census designs (*N* = 6).

The three primary aerial survey issues (nondetection [*N* = 78], counting error [*N* = 27], and species identification [*N* = 10]) were not unique to any specific taxa or environment and 31 papers addressed more than one of the three focal issues of nondetection, counting error, and species identification. Issues related to nondetection were pervasive throughout the entire time period examined; however, the methods used to handle nondetection changed over time. Although counting error and species misidentification were presented as issues in early papers, approaches to rectify these issues were largely missing from the literature until recently.

#### 3.1.2 Case study

During GoMMAPPS surveys, double observers collected 2056 total records during the winter 2018 survey, 1620 in summer 2018, 1706 in winter 2019, and 2584 in winter 2020. We were unable to reconcile double‐observer records for winter 2020 data due to technical difficulties with syncing in‐flight data computer clocks. Recorded flock sizes varied from one individual to thousands across survey events (Maximum counts—winter 2018: 2000; summer 2018: 800; winter 2019: 3200; winter 2020: 500). However, most observations were of single individuals (winter 2018: 71%; summer 2018: 66%; winter 2019: 77%; winter 2020: 74%). The median‐recorded flock size was one individual across all survey time periods while the mean recorded flock size ranged between four and seven individuals for the different survey events. Observers recorded 31–49 unique waterbird species during survey events (winter 2018: 49 species; summer 2018: 34 species; winter 2019: 31 species; winter 2020: 35 species). During winter seasons, the most prevalent waterbird species were northern gannets (*Morus bassanus*) followed by gull and tern (*Laridae*) species, and during the summer seasons, the most prevalent waterbird species were gulls and terns followed by brown pelicans (*Pelecanus occidentalis*).

The issues identified in the literature were present in our waterbird case study, highlighting the high likelihood that these issues are prevalent in most aerial surveys even if they are not reported. We report results from both the literature review and our case study on each of the issues of nondetection, counting error, and species misidentification in the following sections.

### 3.2 Analyses of aerial survey challenges

#### 3.2.1 Nondetection: failure to record individuals when they are present

A majority of papers (76.5%, *N* = 78) identified nondetection as a problem that can lead to undercounting and biased estimates of abundance. Some of the earliest literature (i.e., published before 1985) focused on describing the issue of nondetection but did not offer any comparative analyses. The main source of nondetection described was visibility bias, where animals were not visible because they were concealed or obscured by vegetation or other habitat features. Other sources of nondetection errors that were described in the literature were observer fatigue, sun glare, and poor survey design (i.e., survey units did not cover suitable habitat for target species, or survey timing did not coincide with target species activity patterns). Nearly half of the nondetection literature demonstrated the magnitude of nondetection errors by contrasting different survey methods, such as aerial count surveys versus known‐presence surveys, including telemetry or infrared camera surveys. The remaining nondetection literature developed and introduced methods for estimating and correcting nondetection biases (discussed in further detail below).

In the GoMMAPPS survey data, approximately 36% of observations recorded by one observer were missed by the other observer on the same side of the aircraft (Table 1: No Match across all surveys). Of these missed observations, most were single individuals (77.7%, *N* = 1503), and frequency of missed observations decreased with increasing group size. Of the 36% missed observations, 8% were the result of one observer recording more species present than the other observer (*N* = 158). We note that our “No Match” results include instances where a bird record was available to be counted for one observer and not the other. In certain instances, the movement of the plane resulted in birds flushing from the flight transect, which could have resulted in them having been recorded by one observer (likely front observer) but missed by the other (likely rear observer). Thus, we recognize that our results represent a “worst‐case scenario” for missed observations.

**TABLE 1:** Summary of data matches between two observers recording data on the same side of an aerial survey for each season of the Gulf of Mexico Marine Assessment Program for Protected Species (GoMMAPPS) surveys. We grouped double‐observer records that were recorded within 10 s of each other and classified these records into categories based on the following criteria: Species + Count Match – count and species identification matched between observer records, Generic + Count Match – count and taxonomic family matched between observer records, Species + Bin Match – log10 count bin (i.e., 0, 1–10, 11–100, 101–1000, and 1000+) and species identification matched between observer records (after count matches accounted for), Generic + Bin Match – log10 count bin (i.e., 0, 1–10, 11–100, 101–1000, and 1000+) and taxonomic family matched between observer records (after count matches accounted for), Species Only Match—species identification matched but neither count nor count bin matched between observer records, Generic Only Match—species taxonomic family matched but neither count nor count bin matched between observer records, Mismatch—species did not match between observer records, and No Match—there was no observation from the other observer recorded within 10 s. For the purposes of this study, the identifications of “gull” and “tern” were included in the species‐level identifications described above, and these identifications were pooled under the family Laridae for higher‐level generic identifications

	Winter 2018	Summer 2018	Winter 2019	Total^a^
Species + Count Match	645	458	651	1754 (32.5%)
Generic + Count Match	86	71	85	242 (4.5%)
Species + Bin Match	144	150	111	405 (7.5%)
Generic + Bin Match	84	88	54	226 (4.2%)
Species Only Match	25	17	20	62 (1.2%)
Generic Only Match	11	3	3	17 (0.3%)
Mismatch	339	219	184	742 (13.8%)
No Match	722	614	598	1934 (36.0%)
Total^b^	2056	1620	1706	5382 (100%)

^a^ Row totals are the total of all records in each category across all survey and observers.

^b^ Column totals are the total records for each season across all observers.

We also compared naïve detection probabilities across crew members for each survey event, where we calculated the proportion of records that were matched between pairs of double observers (all but No Match category). Naïve detection probabilities of groups of waterbirds were highly variable among individual observers and across survey events (Table 2). Although pilot‐biologists were responsible for flying aircrafts and collecting survey data simultaneously, their naïve detection probabilities were comparable to those of nonpilot observers (Table 2). Average nondetection errors also differed between the eastern and western halves of our study area with 20% of records classified as No Match for the western half of the study area across winter 2018, summer 2018, and winter 2019 surveys and 58% of records classified as No Match for the eastern half. These results suggest that detection may vary by individual observer, survey event, and spatial location, whether or not that information is included in the analysis of the data.

**TABLE 2:** Naïve detection probabilities for each of the nine observers that participated in the Gulf of Mexico Marine Assessment Program for Protected Species (GoMMAPPS) data collection, calculated as the proportion of records that matched between double‐observer pairs excluding the No Match category. Detection probabilities were highly variable across observers and survey events

Observer	Winter 2018	Summer 2018	Winter 2019	Standard Deviation
Observer 1	—	0.83	—	
Observer 2^a^	0.74	0.83	0.90	0.08
Observer 3	—	0.80	—	
Observer 4	0.82	0.61	0.80	0.12
Observer 5	—	0.49	—	
Observer 6^a^	0.71	0.61	0.70	0.06
Observer 7	0.82	—	0.81	0.01
Observer 8	0.68	—	0.73	0.04
Observer 9	0.65	—	0.67	0.01
Standard Deviation	0.07	0.14	0.08	

Note

Only six observers were used in each survey event. A dash “—” symbol in the survey season columns indicates that the observer did not participate in that survey.

^a^ Pilot biologists.

Methodological and statistical developments for handling nondetection errors have been a major focus of ecological research for decades, and aerial survey research is no exception. Prior to the 1990s, much of the aerial survey literature focused on calculating “correction factors” from “sightability models” (Caughley et al., 1976). These sightability models considered survey variables, such as flight height, flight speed, and observation strip width to calculate a correction factor that was then applied to the aerial count data to correct for visibility biases. Although this method is less common in more recent literature (2000–present), correction factors continue to be used. The primary reason that the use of correction factors has declined is because such models are cumbersome to implement over varying conditions, heterogeneous landscapes, and across multiple species as a different model/correction factor is required for each scenario (Steinhorst & Samuel, 1989). For example, based on the nondetection errors we uncovered in the GoMMAPPS data, we would need to model a correction factor for each region, survey event, and observer, and we would likely need to account for different visibility conditions, as well. Thus, the correction factor approach can become untenable when many factors contribute to nondetection errors.

In the 1990s, other statistical techniques were introduced to formally address issues of nondetection in aerial count data (Quang & Becker, 1997), including distance sampling and reconciled double‐observer methods. In distance sampling (sometimes referred to as “line transect sampling” in the aerial survey literature [Quang & Becker, 1997]), observers move along a transect line and record the distance to detected animals. The recorded distances are used to fit a detection function that describes the change in detection probability as a function of distance from transect and is used to estimate the proportion of animals not detected (Buckland et al., 2001). Reconciled double‐observer methods exploit mark‐recapture methods (often called the “double‐count technique” in early aerial survey literature (Graham & Bell, 1989), where two observers independently record the number of detected animals and agree on which animals were detected by both observers. The first observer “marks” and “releases” certain animals while the second observer “recaptures” the animals. This creates a two‐occasion capture history that can be used to estimate the number of missed animals (Graham & Bell, 1989). These methods are an improvement over use of correction factors because they allow researchers to model detection as a dynamic variable across heterogeneous environments and visibility conditions, as well as estimate uncertainty around detection probability (Walter & Hone, 2003).

In the last decade, researchers have combined methods for estimating detection from double‐observer mark‐recapture and distance sampling into a single model (Burt et al., 2014). This approach uses the strengths of both distance sampling and mark‐recapture sampling to fit a detection function where the shape of the function is estimated with distance sampling methods and the intercept of the function is estimated using the mark‐recapture data (Laake et al., 2008). Mark‐recapture distance sampling thus relaxes the assumption of distance sampling that all individuals on the transect line are detected, which is often violated in aerial surveys. A double‐observer protocol, as required for mark‐recapture sampling, can be prohibitively expensive because two observers are needed to record the same data. Thus, these approaches to correct for nondetection may not be feasible under all survey scenarios. In such cases, researchers should consider whether estimates of detection probability are feasible given the sampling conditions and operational budget.

Distance sampling was not feasible in the GoMMAPPS surveys because there was limited time available to record all the necessary data from mixed‐species flocks, which is frequently the case for highly mobile species. In addition, when groups are loosely aggregated, crossing over multiple distance bands or expanding outside the sampling area, as was often the case on our surveys, it is unclear from where the distance measurement should be taken, and this becomes especially burdensome when groups contain multiple species. Double‐observer mark‐recapture sampling was also impractical with the GoMMAPPS surveys again because of the limited time available to reconcile which individuals were seen (or missed) by both observers due to the speed of the planes and fast mobility of waterbirds. Thus, the GoMMAPPS project reveals that many of the analytical approaches designed to account for nondetection may be impossible to implement broadly. However, post hoc data processing allowed us to reconcile double‐observer counts that were recorded within 10 s of each other. The reconciled data could then potentially be used to fit mark‐recapture models to estimate detection probability, if the assumptions made to simplify data collection (e.g., a temporal cutoff for matching records) are tenable. Using the reconciled double‐observer data, we found differences in detection between survey regions. Thus, a covariate to capture this spatial heterogeneity would be useful to estimate indices of abundance in our case, particularly in a region like the northern Gulf of Mexico where levels of anthropogenic activity vary from west to east. When sampling methods that allow for estimating detection probability are infeasible, researchers should carefully consider observational variables that may affect detection and include those in their models when estimating indices of abundance (Johnson, 2008). However, in doing so, researchers should recognize a potential loss in clarity of inference when variables that affect abundance and detection are both present in a single model (i.e., not separated in a hierarchical framework).

#### 3.2.2 Counting Error: failure to correctly enumerate group size

Most aerial survey research treats issues of counting error as a nondetection problem. However, about one quarter of the papers (*N* = 27) addressed counting error distinctly from nondetection. When possible to assess, researchers have found that observers undercount group size on average, leading to underestimates of abundance (Frederick et al., 2003; Gerrodette et al., 2019). Many aerial survey efforts focus on species that often occur in groups, including wintering waterfowl, seabirds, wading birds, ungulates, and cetaceans among others. Aerial counts of animals are usually obtained by trained in‐flight observers or by collection and analysis of aerial photos and videos captured by on‐board cameras (Chabot & Francis, 2016; Žydelis et al., 2019). Although widely used, in‐flight observer counts are often biased (Caughley, 1977; Jolly, 1969), with variability among observers and a tendency to underestimate group size (Chabot & Francis, 2016), particularly for large groups (Buckland et al., 2012; Frederick et al., 2003).

**FIGURE 3:**
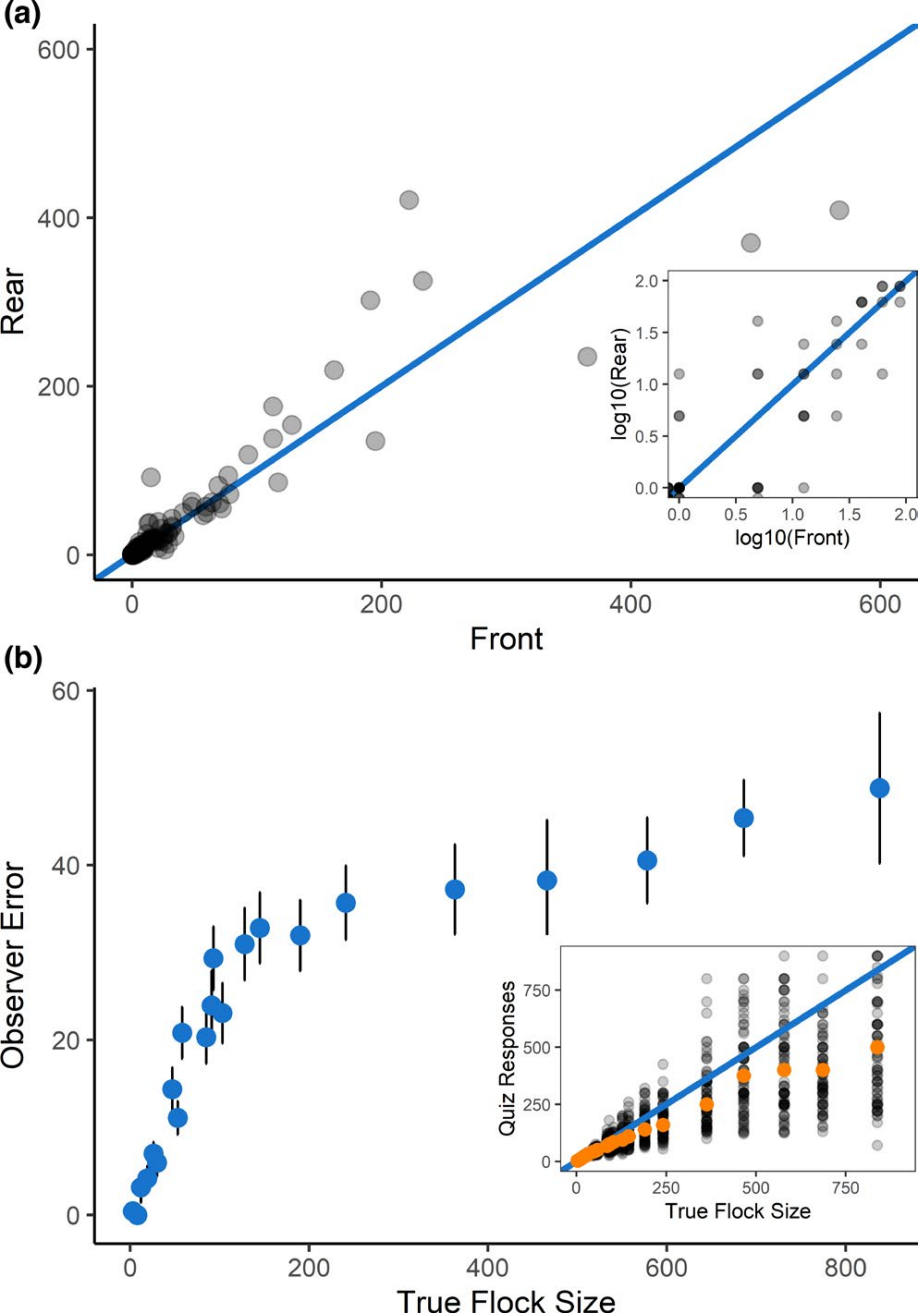
(a) Counts of waterbirds from front and rear same‐side observers, shown for the winter 2018 Gulf of Mexico Marine Assessment Program for Protected Species (GoMMAPPS) survey. The inset figure shows the log10 of flock counts <100 of waterbirds from front and rear same‐side observers. Blue lines show 1:1 lines. (b) Absolute value of the percent difference in respondents’ counts and the true flock size for each of the 22 quiz images. Points plotted are the mean absolute value of the percent difference in respondent counts and true flock sizes. Error bars are 95% confidence intervals. The inset figure shows quiz responses (black points) from 78 online counting quiz respondents for each of the 22 quiz images. Orange points show the median response value for each of the 22 quiz images. The blue line is a 1:1 line; observer responses plotted above this line are overcounts of the true flock size, and observer responses plotted below this line are undercounts

In the GoMMAPPS survey data, flock counts varied between same‐side observers, with the magnitude of differences between front‐ and rear‐observer counts increasing with flock size (Figure 3a). Double‐observer records indicated that roughly 33% of observers’ counts (across all species) matched across records from all surveys (*N* = 1996; ~85% of which consisted of single‐individual count matches). Thirty‐five percent of double‐observer counts matched for flocks with five individuals or fewer across all possible species in a flock (*N* = 4946), and approximately 10% of double‐observer log10 count bins matched when exact counts did not match. For flock sizes between six and 30 individuals (*N* = 319), approximately 7.5% of double‐observer counts matched, and approximately 33% of double‐observer log10 count bins matched when exact counts did not match. Approximately 8.5% of double‐observer count records matched for flock sizes greater than 30 individuals (*N* = 117), and approximately 32% of double‐observer log10 count bins matched when exact counts did not match. Counts >30 individuals may have had slightly more agreement between double observers than smaller group sizes due to similar rounding tendencies when large groups were encountered. Binning counts into a log10 categorical scheme resulted in substantially more agreement between double‐observer records than comparing exact counts alone (49% agreement versus 37% agreement, respectively), particularly for flock sizes >5 individuals.

Our online quiz revealed that even trained observers have difficulty correctly enumerating flock size from images taken during aerial surveys of waterbirds (Figure 3b, Appendix S2, Figures A3–A5). At large flock sizes (200–1000), observer counts ranged from 40% to 150% of the true flock size, which is consistent with previous studies (Frederick et al., 2003). On average for the large flocks (>100), observer counts were 35–48% of the true flock size. However, even at small flock sizes (<100), mean observer errors were as high as 30%. Observers most frequently under‐counted flock sizes, a tendency that increased with flock size with 50–70% of all observers underestimating flock size some or all the time when the true size was above 30 individuals. Observer experience and confidence in counting skills had no effect on observer ability to correctly enumerate flock size in the online quiz (Appendix S2, Figures A6–A7).

As most aerial survey research treats counting error as a nondetection issue, the solutions for handing nondetection errors are generally applicable to counting errors. However, recent advances in hierarchical modeling have made it possible to partition nondetection errors from counting errors. Clement et al. (2017) combined a mark‐recapture distance sampling model with an N‐mixture model (Royle, 2004) to separately account for nondetection and counting errors. Under this approach, observers independently recorded counts of observed groups in addition to the detection history and distance data collected for a mark‐recapture distance sampling model. Combining the three sampling methods into a single hierarchical model allows for unbiased abundance estimates when observers imperfectly detect individuals due to nondetection errors, as well as counting error (Clement et al., 2017). A limitation to this model is that it requires a double‐observer protocol, which may be costly or impractical for some survey efforts, and it also requires distance sampling, which is not feasible in all survey situations (such as the GoMMAPPS surveys).

Another potential solution for handling counting errors is to use ordinal modeling (Guisan & Harrell, 2000). In this approach, count data are binned into categories (e.g., 0, 1–10 individuals, 11–50 individuals), and the probability of obtaining a certain category is then modeled instead of the counts directly (Guisan & Harrell, 2000). The appropriate bin breaks may be based on a log scale or another scale based on magnitude of observer error (Figure 3) or where natural breaks occur in the data (Valle et al., 2019). Modeling‐binned count data rather than the counts themselves may alleviate potential concerns regarding inferences based on counts with errors and allow for the estimation of uncertainty around the probability of ordinal classifications (Fitzgerald et al., 2021). Additionally, if ordinal modeling approaches are comparable to or better than the typical approach of using a count distribution (such as the negative binomial) to model abundance (Zipkin et al., 2014), collecting count data on a categorical scale may limit the training and time needed to for data collection. Count data may also be binned after field data collection if concerns arise regarding accuracy of recorded counts.

#### 3.2.3 Misidentification: failure to correctly identify individuals

Wildlife survey data are often analyzed without consideration of species identification errors, despite evidence that identification errors occur regularly (Conn et al., 2013). Indeed, only 10 out of the 102 (~10%) papers directly dealt with species misidentification. Papers that reported issues associated with species misidentification tended to focus on small‐ or medium‐bodied animals that are difficult to clearly identify or even detect from the air (Greene et al., 2017; Lamprey et al., 2020). One paper addressed difficulties with age and sex classification in elk (*Cervus canadensis*); like species identification, age, and sex classification also requires observers to discern small details from the survey aircraft (Bender et al., 2003).

Although the GoMMAPPS survey observers were trained in waterbird species identification, our double‐observer data indicated that only ~41% of the observations recorded by both observers contained matching species identifications (Table 1: species + count match, species + bin match, and species only match categories). In addition, GoMMAPPS observers had difficulty discerning individual gull and tern species due to their small body sizes, speed of the planes, and often indiscernible features (e.g., similar plumage characteristics, body‐size, or bill shape); thus, higher‐level gull and tern identifications (e.g., gull, tern, or larid spp.) were used when definitive species identifications could not be made (~85% of all gull and tern records across survey events). Generic identifications, including individuals identified by double observers as different species within the same taxonomic family (e.g., white‐winged scoter [*Melanitta deglandi*] versus black scoter [*Melanitta americana*]) or individuals that were not identified to species‐level (except for gulls and terns), comprised ~9% of the total records (Table 1: generic + count match, generic + bin match, and generic only match categories). Mismatched records, including individuals identified as different species by the two observers (where taxonomic family also did not match between double‐observer records), comprised ~13% of the total records (Table 1). It is possible that some of these records are likely to be detection errors rather than misidentification errors, as we could not separate these issues. Nevertheless, given the findings of our study, it appears that species identification errors are likely to be present and possibly pervasive in multispecies aerial survey datasets, especially when similar species (e.g., similar in body size and/or coloration) co‐occur as they often do in waterbirds.

Auxiliary data that contain species‐level (or sex/age class) records are generally needed to correct identification errors. For most studies in our literature review, researchers were only able to report that identification errors existed because they had access to other, independent survey data in addition to the aerial survey data (e.g., ground‐based [Laursen et al., 2008, Greene et al., 2017, Lamprey et al., 2020] and vessel‐based [Johnston et al., 2015] surveys). However, auxiliary data are rarely available because it is costly and time consuming to obtain as it requires a second, simultaneous surveying effort. Furthermore, secondary surveys may suffer from the same errors present in aerial survey data. Because of misidentification errors, it may be inappropriate to model individual species, sex, or age class counts when identifying features are difficult to distinguish and secondary sources of data are unavailable.

No secondary sources of data were available to complement the GoMMAPPS aerial survey data that could be used to correct for potential misidentification errors. However, when we aggregated species records by higher‐order taxonomic classifications (i.e., family), we found that the records complementarily identified by both observers (front and rear) increased by approximately 10% (Table 1: generic + count match, generic + bin match, and generic only match categories). This suggests that analyzing data at higher taxonomic levels, rather than species, may be a reasonable approach to overcome identification issues when similar species co‐occur if species‐level identification is not a primary goal.

## 4 SYNTHESIZING SOLUTIONS: A PATH FORWARD

Our literature review and empirical case study reveal that issues of nondetection, counting error, and species misidentification are prevalent in aerial survey count data. The extent to which each of these issues may bias inferences depends on the unique circumstances of individual survey efforts, including the frequency and severity of the errors as well as the goals of the survey and the subsequent analyses of the data. The ideal approach for mitigating potential biases from aerial survey data will vary based on the specific questions asked and which issue(s) are likely to occur with the particular survey conditions. A good first step when designing an aerial count survey is to determine which issues are probable under given survey conditions. For example, if the aerial survey targets a single, solitary species, counting and species misidentification errors are unlikely to present serious issues for inference, leaving nondetection as the only major source of bias. However, when multiple, similar‐looking species that aggregate are targeted, such as in our GoMMAPPS case study, all three detection errors should be carefully considered. Prior to data collection, a simulation study can help determine the extent to which nondetection, counting, and misidentification errors may bias estimates under various survey conditions, revealing where and when survey effort would be best utilized (e.g., focusing on improving species identification versus nondetection errors) to minimize effects on abundance and covariate inferences. In any case, the likelihood of encountering these issues should be considered prior to designing an aerial sampling scheme to minimize potential errors or at least understand when and where they might occur.

Sampling errors can be partially mitigated during survey planning, data collection procedures, and/or data analysis stages of a project. The appropriate methods for handling various sources of bias will depend on the stage at which these issues are considered. If the potential for nondetection, counting, and misidentification errors is considered during study development, steps can be taken to design a survey that can both identify and measure these errors while taking logistical considerations into account. Recognizing conditions that are likely to present data issues can help researchers identify where and when resources may best be used to maximize count data quality and when auxiliary sources of data may be necessary to address research goals. Considering the many methods presented in the literature and our own experience with waterbird aerial surveys, we consolidate the methods presented above into a few recommended approaches for handling nondetection, counting, and identification errors. Our recommendations are organized across the different stages of survey implementation including survey planning, sampling methodology, and modeling approach. We also suggest potentially exciting future directions in aerial survey research and methods.

### 4.1 Survey planning

Selecting an appropriate sampling framework for particular research question(s) or management objective(s) is paramount for choosing an effective study design that will result in reliable inferences. However, aerial surveys must balance a number of logistical and practical considerations with the scientific goal(s) of the study. Logistical considerations, such as defining the spatial extent of sampling and determining the appropriate configuration of aerial sampling units influence survey cost and efficiency and contribute to what sampling methodologies are feasible (Caughley, 1977; Gibbs et al., 1998). Standardizing sampling methodologies across survey events, including the survey design as well as data collection protocols, is important to ensure that indices of abundance or distribution of species are comparable across years/seasons.

These design considerations also influence the types of research questions that are possible to address. Understanding the effects of environmental variables on species abundance requires a great deal of survey data, and if this is the goal, researchers should prioritize sampling a range of environmental conditions many times (Zipkin et al., 2015). However, if the goal of the survey is to estimate abundance of a species (or a group of species), researchers may consider using probabilistic sampling strategies rather than stratifying sampling units across the full range of environmental variables of interest. In many cases, aerial surveys are used to estimate indices of abundance or distribution that are used to track changes over time, and if this is the goal of the survey, researcher efforts should focus on maintaining consistency in design, personnel, and protocols over time to minimize observer errors related to changes in the survey. When designing an aerial survey, researchers are encouraged to carefully consider their research goals and the extent to which survey design can be used to either mitigate or elucidate nondetection, counting, and misidentification errors.

### 4.2 Sampling methodology

Our literature review revealed that distance sampling is the most popular framework for collecting aerial count data and modeling abundance with a detection probability. However, we suggest this approach only be used when observers have adequate time to record distances (or distance bands) and when the survey targets a small number of species. One solution could be using high resolution photography or video in addition to or instead of in‐flight observers which would allow for a number of different analytical methods for estimating abundance and detection probability. However, in the presence of other errors (e.g., counting and species identification errors), estimating absolute abundance may be misguided as relative abundance indices may be the only obtainable parameter.

The double‐observer method can be used to reconcile disparate observer data during data collection if observers work together to agree on what was observed (Quang & Becker, 1997). However, in the GoMMAPPS case study, the speed of the aircraft and frequency of observations presented logistical limitations for reconciling in‐flight double‐observer data. Additionally, employing a second observer to record duplicate data may be prohibitively expensive for some survey efforts. Thus, we emphasize the importance of standardizing survey design and sampling protocols across survey efforts to minimize potential biases related to differences in procedures among surveys. However, use of a second observer (even on a limited basis), to estimate detection factors and counting errors and/or assist with species identification can be very beneficial to determining the rate and nature of errors. When estimating detection errors explicitly is intractable, researchers may opt to instead estimate relative abundance using generalized linear models by incorporating covariates that affect detection into models of abundance.

### 4.3 Modeling

Despite survey training, numerous studies, including our own waterbird work, have shown that in‐flight observers often undercount group size, especially for large, aggregated groups, and misidentify species when multiple species are present. Without auxiliary data, such as a simultaneous survey effort (e.g., ground‐based or vessel‐based sampling) or a double‐observer protocol, it is impossible to identify that these errors are present. When group sizes are very small (<10 individuals) and only a single species or obviously dissimilar species are targeted (e.g., African elephant [*Loxodonta africana*] versus African buffalo [*Syncerus caffer*] [Greene et al., 2017]), these errors may be limited; however, outside of these circumstances, it is likely that counting and misidentification errors are not only present but also prevalent. Nevertheless, despite these issues, aerial surveys have often been used to identify significant changes in population sizes through time as well as to elucidate important environmental relationships. In these cases, it is assumed that the effect sizes of covariate relationships are larger than the errors encountered during data collection, yet when these errors are substantial and variable across surveys, abundance indices may not be accurate and important covariate relationships may be missed.

An obvious, albeit perhaps unsatisfying suggestion for addressing species misidentification and counting errors, is to pool data. Although grouping species records by taxonomic group or foraging guild dilutes information contained in the data, it may alleviate some species misidentification errors when similar species are targeted. Pooling count data to create binned categories decreases the resolution of available data but may more accurately reflect the true uncertainty regarding the precision of the survey count data (Guisan & Harrell, 2000; Valle et al., 2019). Our GoMMAPPS analyses suggest that binning counts is beneficial for group sizes as small as 6–30 individuals and certainly for group sizes reaching hundreds or thousands of individuals. Although ordinal modeling is used relatively infrequently in ecology, this framework offers a promising alternative to modeling exact counts and can reflect uncertainty in count data when counting errors may be present. If species‐level inferences are required, researchers could explore data integration with publicly available datasets (e.g., eBird, iNaturalist). Data integration, or modeling that incorporates multiple, dissimilar data types, (e.g., count data and presence/absence data) can yield more detailed information about a process of interest, including more accuracy and precision in estimates, than an analysis using a single data source (Zipkin et al., 2019).

### 4.4 Digital data collection and future directions

The last decade of aerial survey research has seen a rise in digital data collection methods, including photography and video collected by drones and unstaffed aerial vehicles (Corcoran et al., 2021; Nowak et al., 2019). These technologies have the advantage of being less expensive than traditional staffed flights as well as being safer for research personnel as they do not require in‐flight observations. However, a drawback to unstaffed aerial vehicles is that it is not possible to cover as large of a spatial area as quickly as can be done in a traditional staffed flight. Nevertheless, photo and video observations typically produce higher quality abundance and density estimates than traditional in‐flight observer methods (Buckland et al., 2012; Chabot & Francis, 2016). After data are collected, photographs and videos may be reviewed by numerous observers which can allow researchers to utilize a number of different methods for estimating detection probability, as well as identifying counting and identification errors. However, despite these advantages, this technology is not immune to the previously discussed issues. Manual image or video classification is subject to the same human errors of nondetection, counting error, and especially species misidentification that in‐flight observers experience (Chabot & Francis, 2016). Although photos and videos may be proofed multiple times, this is time‐consuming and potentially costly. High resolution photography and videography is undoubtedly helpful in resolving counting errors, but imagery must be of high enough quality that distinguishing features can be discerned to differentiate among similar species.

Digital object classification (i.e., machine learning) offers a promising way forward for handling the time‐intensive data processing required of digital data collection. Methods for automating object classification have improved in recent years and are already useful for reducing nondetection and counting errors (Torney et al., 2019), but automated species identification is more challenging (Chabot & Francis, 2016; Villon et al., 2020). Future work on digital object classification presents an opportunity to engage the public to help classify images that can be used as training data for classification algorithms (Torney et al., 2019), broadening the impact of research beyond the study system itself (Adler et al., 2020). Although digital methods may help to combat some of the human errors observed in the literature (including our own work), these technologies may also suffer some of the same shortcomings as count data collected by in‐flight observers. Thus, the suggestions presented in this paper should be useful for combatting errors in count data collected both by human observers and digital methods.

## 5 CONCLUSIONS

Imperfect detection can manifest as nondetection, counting error, and species misidentification, and all these sources of error should be considered when collecting and analyzing aerial survey data. Although counting error and species misidentification have received comparatively limited attention (and thus fewer solutions) relative to nondetection, errors generated by all three sources are present and likely prevalent in aerial survey count data. Ignoring these errors or neglecting to address them explicitly could lead to biased or misleading inferences. Researchers should be aware that these issues exist and take measures to combat them during the design, data collection, and analysis stages of a study. Recognizing the conditions that can lead to data collection errors can allow researchers to allocate resources toward minimizing potential errors or invest more resources toward goals with fewer perceived challenges.

## CONFLICT OF INTEREST

The authors declare that they have no known competing financial or personal relationships that could have influenced the work reported in this paper.

## AUTHOR CONTRIBUTIONS

**Kayla L. Davis:** Conceptualization (lead); Data curation (lead); Formal analysis (lead); Investigation (lead); Writing – original draft (lead). **Emily D. Silverman:** Formal analysis (supporting); Methodology (equal); Writing – review & editing (equal). **Allison L. Sussman:** Data curation (equal); Writing – review & editing (equal). **R. Randy Wilson:** Funding acquisition (lead); Project administration (lead); Resources (equal). **Elise F. Zipkin:** Conceptualization (lead); Resources (equal); Supervision (equal); Writing – review & editing (equal).

### OPEN RESEARCH BADGES

This article has earned an Open Data Badge for making publicly available the digitally‐shareable data necessary to reproduce the reported results. The data is available at https://doi.org/10.5281/zenodo.6038240.

## Supporting information

Appendix S1

Appendix S2

## Data Availability

Data and code are available on Github (https://github.com/zipkinlab/Davis‐et‐al_Aerial‐Survey) and archived on Zenodo Digital Repository (Davis, 2021). https://doi.org/10.5281/zenodo.6038240.
