# Supplementary material for: Errors in aerial survey count data: Identifying pitfalls and solutions
Source: Ecol Evol. 2022 Mar 18;12(3):e8733. doi: 10.1002/ece3.8733 (PMC8931709; doi:10.1002/ece3.8733)

Appendix 2. Flock Size Quiz

##

## Flock Size Quiz Methods

We designed an online flock counting quiz using Qualtrics survey software. The design and content of the quiz were adapted from the U.S. Fish and Wildlife Service Aerial Observer Training and Testing Resources (<https://www.fws.gov/waterfowlsurveys/>). We distributed the quiz via email to trained observers in the U.S. Fish and Wildlife Pilot Biologist Program and biologists with no aerial survey experience. The quiz consisted of background questions regarding respondents’ level of experience conducting aerial bird surveys (Expert [collected data for 5+ aerial bird surveys], Intermediate [collected data for 2-5 aerial bird surveys], Novice [collected data for 1 aerial bird survey], No Experience [collected data for 0 aerial bird surveys]) and confidence in their flock counting skills (High, Medium, Low). The flock counting portion of the quiz consisted of two practice images and 22 timed quiz images of known-size flocks. Each image was displayed for 10 seconds before it disappeared, and the quiz automatically advanced to a question asking “How many birds were in the image?” The flock sizes in presented images ranged from 3-838 individuals (Figure A1). We chose to feature images of relatively small flocks (<100 inds) most heavily because the GoMMAPPS field data indicated that most of the flocks observed were in this size range (Figure A2), and previous studies have focused on large flocks (200-6000 inds; Frederick et al. 2003). Given the frequency of small flocks in our field data and the relative lack of information on observer counting errors at small flock sizes, we deemed it important to include more small flock images in our quiz set than large flock images.

## Results

After distributing the quiz via email, we collected responses for three weeks. We received 89 responses in total, and we were able to use 78 of those for further analysis. The 11 quiz responses that we discarded were either test responses that were collected before the official survey distribution window or were incomplete. We received quiz responses from respondents with various levels of experience conducting aerial surveys (Table A1) and confidence in their flock counting skills (Table A2). The actual flock sizes, mean respondent count, and mean absolute percent difference in respondent count and actual flock size are given in Table A3.

As we expected, we found that observer error increased with flock size (Figure A3). The percent difference in quiz respondent counts and the true flock size were similar to what Frederick et al. 2003 observed. For flock sizes >200 individuals, respondents were, on average, 40-50% off in their counts from the true flock size values (Figure A4). Even at relatively small flock sizes (<100 individuals), average respondent counts were as much as 30% different from the true flock sizes (Figure A4). Most observers underestimated flock size (Figure A5). Across all flock sizes presented (3-838), 62% of responses underestimated the true flock size. Underestimation was most apparent for flock sizes of ~30 individuals or more with 50-70% of responses underestimated for all images with flock sizes of 30 or greater (Figure A6).

There was little difference in observer error among respondents across the experience levels (Figure A7). However, respondents who ranked themselves with intermediate levels of confidence in their flock counting skills performed slightly better than respondents with high or low levels of confidence; although, this difference was not significant (Figure A8).

**Tables**

Table A1. The number of quiz respondents who self-identified in each experience category representing their level of experience conducting aerial surveys for counts. The experience categories were based on the number of aerial bird surveys the respondent had completed: Expert (collected data for 5+ aerial bird surveys), Intermediate (collected data for 2-5 aerial bird surveys), Novice (collected data for 1 aerial bird survey), No Experience (collected data for 0 aerial bird surveys).

| Experience Level | Number of Respondents |
| --- | --- |
| Expert | 38 |
| Intermediate | 20 |
| Novice | 10 |
| No Experience | 10 |

Table A2. The number of quiz respondents who self-identified in each confidence category representing their level of confidence in their own flock counting skills.

| Confidence Level | Number of Respondents |
| --- | --- |
| High | 14 |
| Intermediate | 48 |
| Low | 16 |

Table A3. True flock sizes of each quiz question (True), the mean and standard error of respondent counts, and the absolute percent difference and standard error of percent difference in respondent counts and the true flock size.

| Question Number | True | Mean Count | SE Count | Absolute % Diff | SE % Diff |
| --- | --- | --- | --- | --- | --- |
| Q3 | 3 | 3.01 | 0.01 | 0.00 | 0.00 |
| Q12 | 8 | 8.00 | 0.00 | 0.00 | 0.00 |
| Q15 | 12 | 11.97 | 0.11 | 0.03 | 0.01 |
| Q9 | 19 | 19.23 | 0.16 | 0.04 | 0.01 |
| Q16 | 26 | 24.92 | 0.24 | 0.07 | 0.01 |
| Q10 | 30 | 30.08 | 0.32 | 0.06 | 0.01 |
| Q14 | 47 | 42.32 | 0.81 | 0.14 | 0.01 |
| Q18 | 53 | 51.44 | 0.82 | 0.11 | 0.01 |
| Q7 | 58 | 47.10 | 1.07 | 0.21 | 0.02 |
| Q19 | 85 | 76.55 | 2.15 | 0.20 | 0.02 |
| Q1 | 91 | 76.79 | 2.66 | 0.24 | 0.02 |
| Q4 | 93 | 69.81 | 2.37 | 0.29 | 0.02 |
| Q8 | 103 | 85.12 | 2.57 | 0.23 | 0.02 |
| Q2 | 128 | 100.04 | 4.25 | 0.31 | 0.02 |
| Q5 | 145 | 110.95 | 4.84 | 0.33 | 0.02 |
| Q13 | 190 | 154.10 | 6.89 | 0.32 | 0.02 |
| Q17 | 241 | 174.32 | 8.09 | 0.36 | 0.02 |
| Q22 | 363 | 278.12 | 15.47 | 0.38 | 0.03 |
| Q21 | 466 | 407.90 | 25.48 | 0.39 | 0.04 |
| Q11 | 578 | 447.19 | 26.51 | 0.40 | 0.03 |
| Q20 | 686 | 435.03 | 26.34 | 0.46 | 0.02 |
| Q6 | 838 | 584.38 | 51.91 | 0.48 | 0.04 |

**Figures**


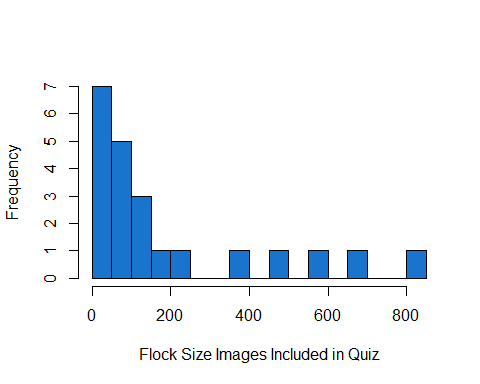


Figure A1: Histogram of known flock size images (true sizes) included in the flock size counting quiz. Flock sizes ranged from 3-838 individuals.


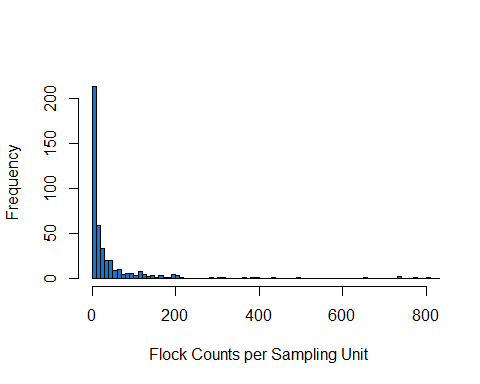


Figure A2: Histogram of flock size counts recorded per sampling unit during winter GoMMAPPS surveys (2018-2020).


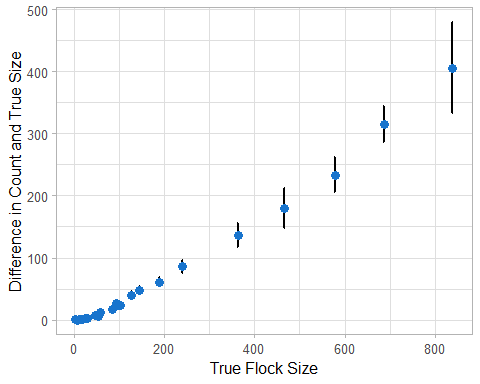


Figure A3: Absolute value of the difference in respondents’ counts and the true flock size for each of the 22 quiz images. Points show the mean absolute values and error bars are 95% confidence intervals. Differences in respondents’ counts and the true flock size increased with flock size.


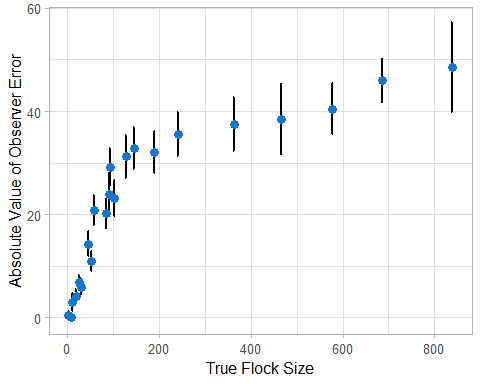


Figure A4: Absolute value of the percent difference in respondents’ counts and the true flock size for each of the 22 quiz images. Points show the mean absolute values and error bars are 95% confidence intervals. Respondent errors increased with flock size and were as high as approximately 50% of the true flock size.


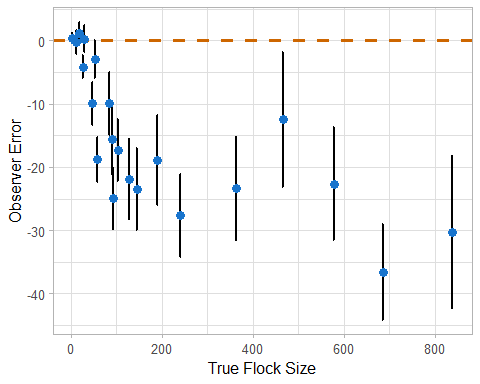


Figure A5: Percent difference in respondents’ counts and the true flock size for each of the 22 quiz images. Points show the mean absolute values and error bars are 95% confidence intervals. In most cases, respondents underestimated flock size, and this tendency to underestimate increased with flock size.


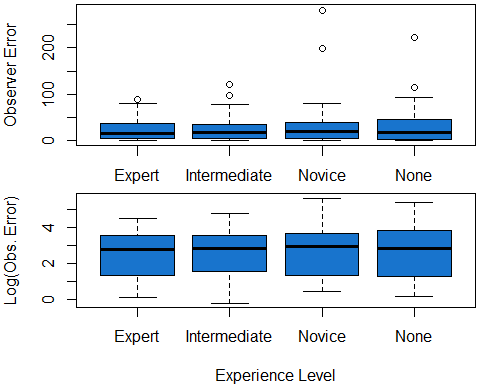


Figure A6: Boxplots of observer error and log of observer error (absolute value of percent difference in respondent count and true flock size) for respondents in each of the four experience categories. There was virtually no difference in observer error across experience levels.


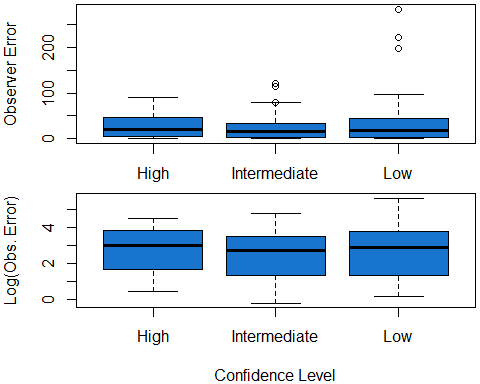


Figure A7: Boxplots of observer error and log of observer error (absolute value of percent difference in respondent count and true flock size) for respondents in each of the three confidence level categories. There was no difference in observer error across confidence level categories; although, respondents who self-identified as having intermediate levels of confidence in their counting skills performed slightly better than respondents with low and high levels of confidence in their counting skills.

**Images used in flock size quiz in the order presented**

Q1. 91


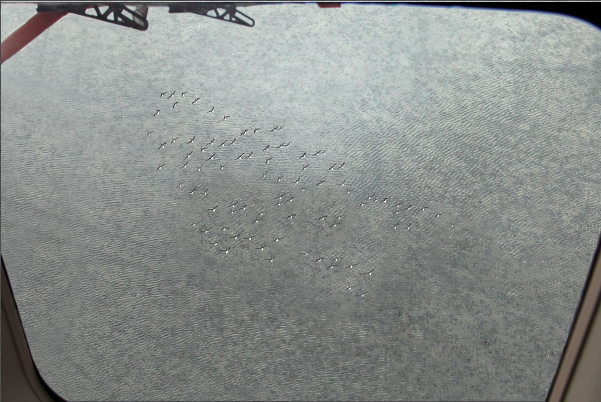


Q2. 128


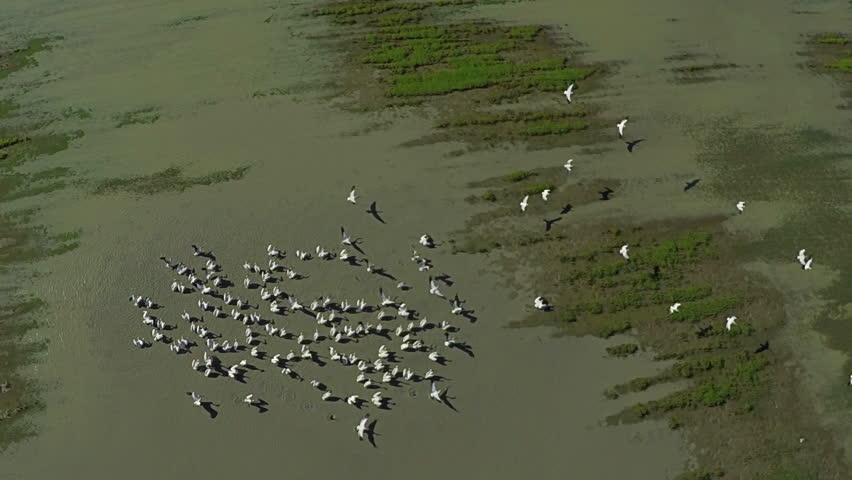


Q3. 3


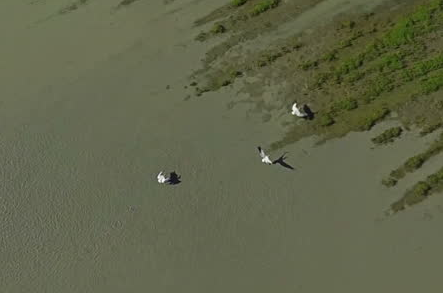


Q4. 93


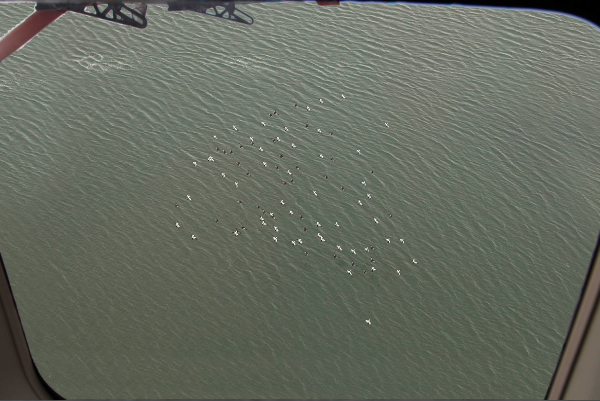


Q5. 145


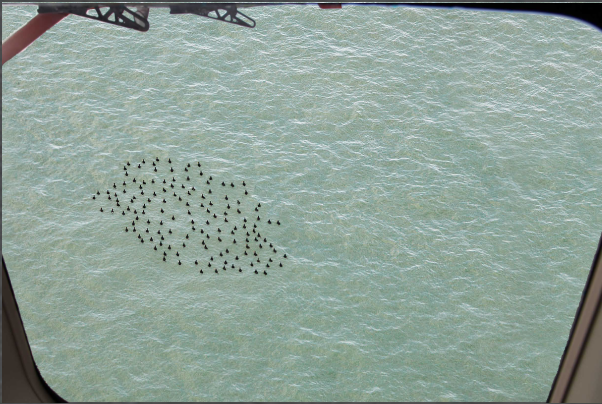


Q6. 838


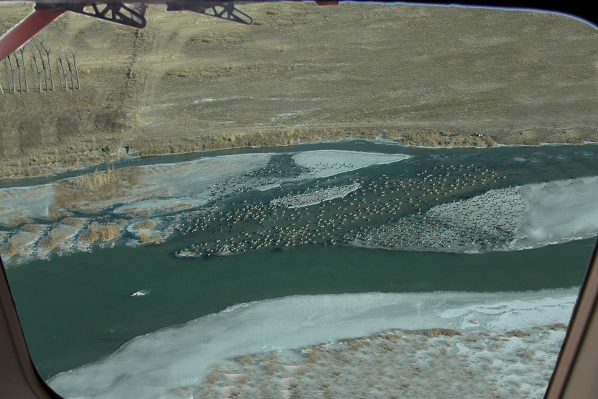


Q7. 58


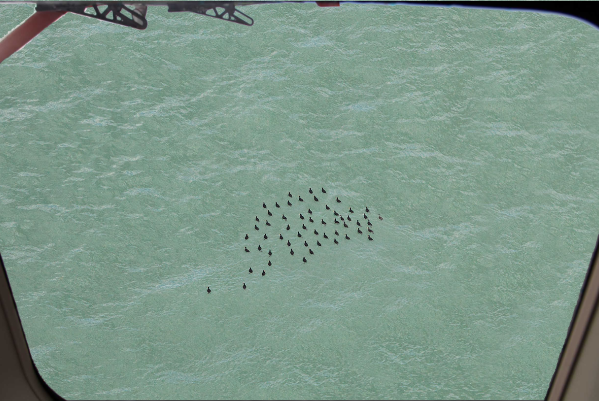


Q8. 103


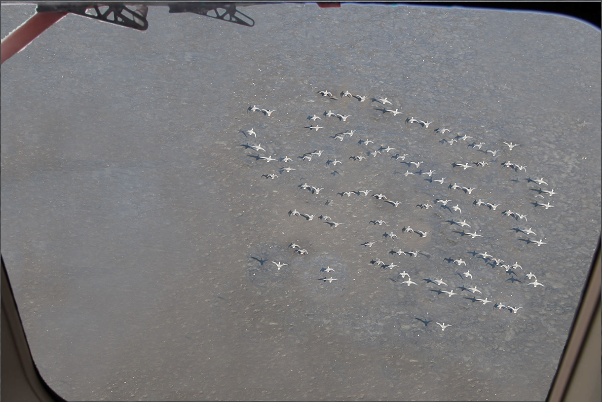


Q9. 19


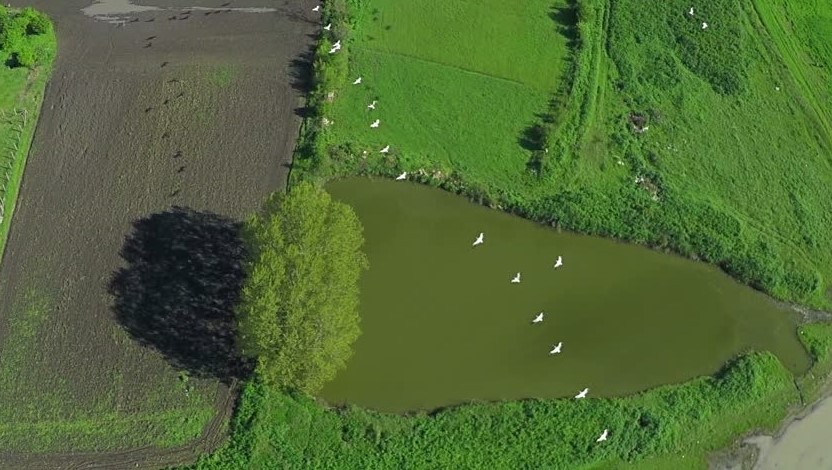


Q10. 30


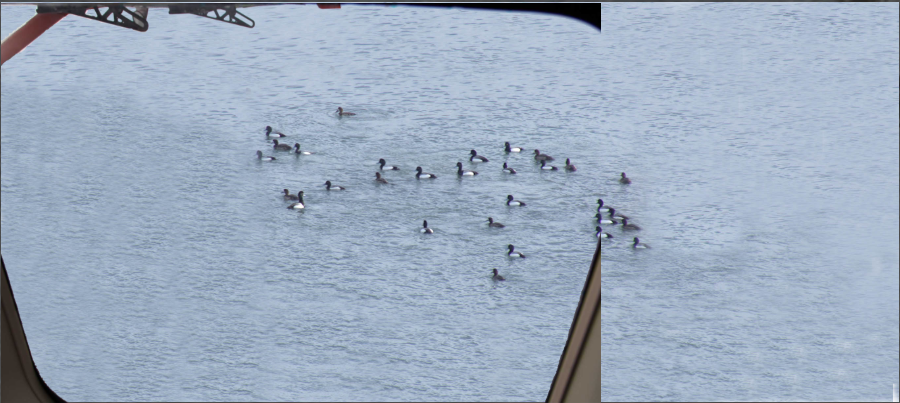


Q11. 578


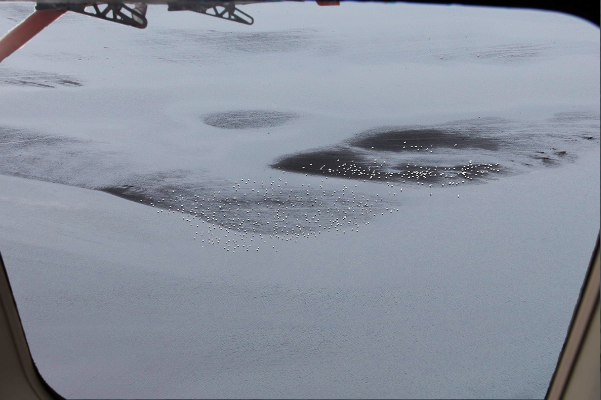


Q12. 8


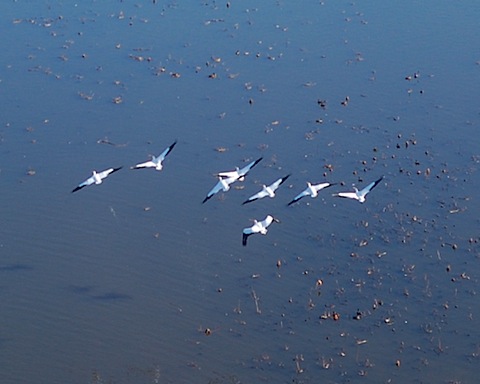


Q13. 190


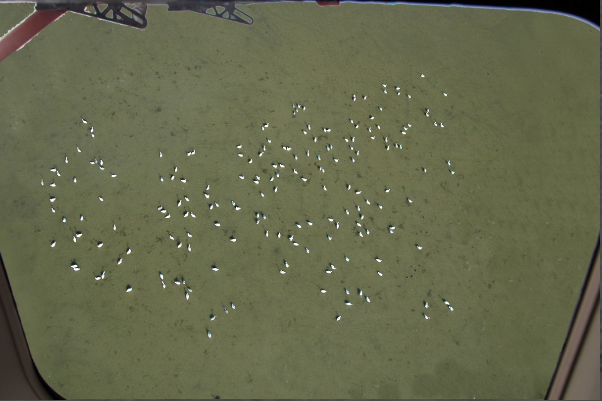


Q14. 47


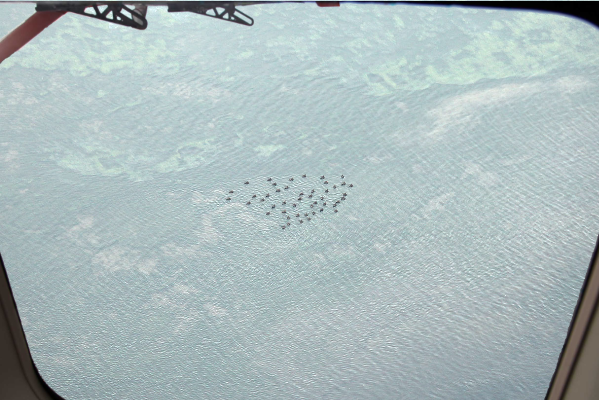


Q15. 12


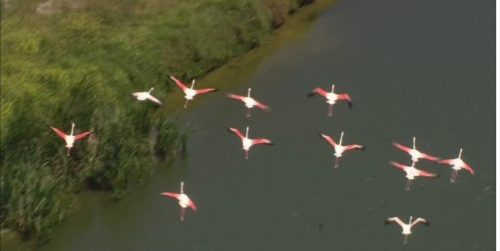


Q16. 26


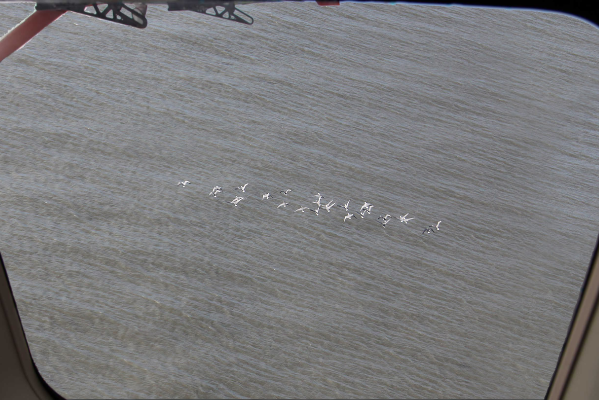


Q17. 241


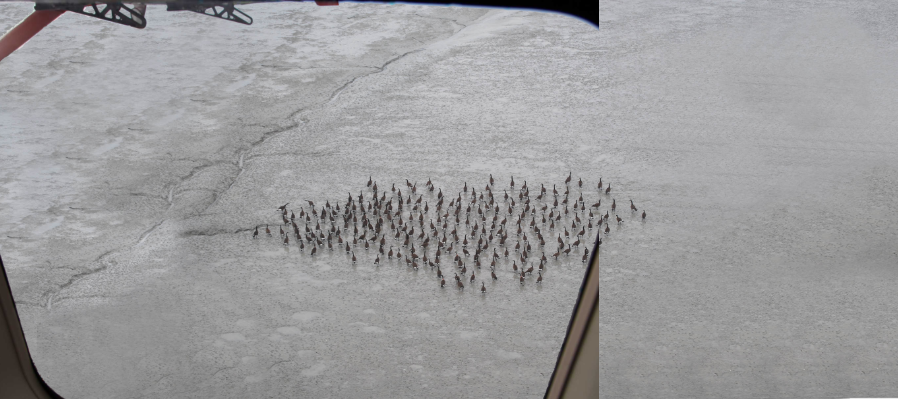


Q18. 53


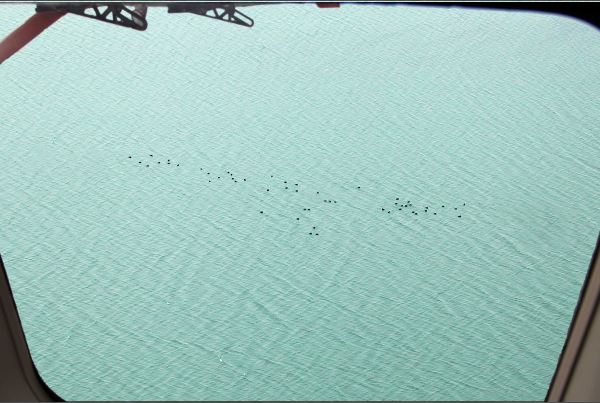


Q19. 85


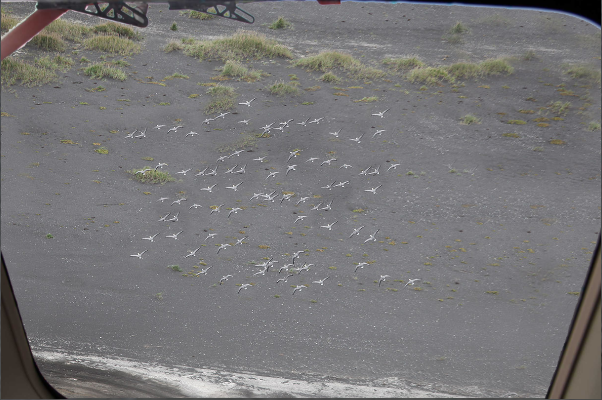


Q20. 686


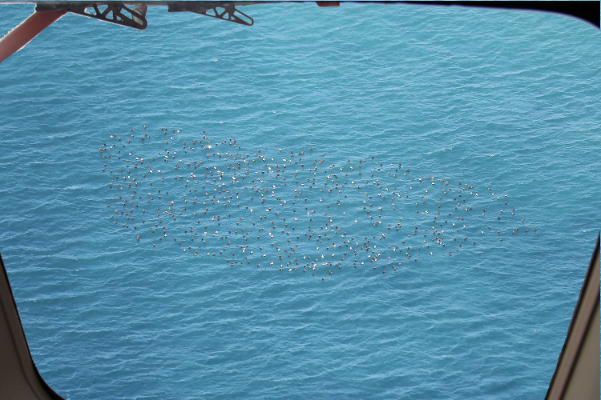


Q21. 466


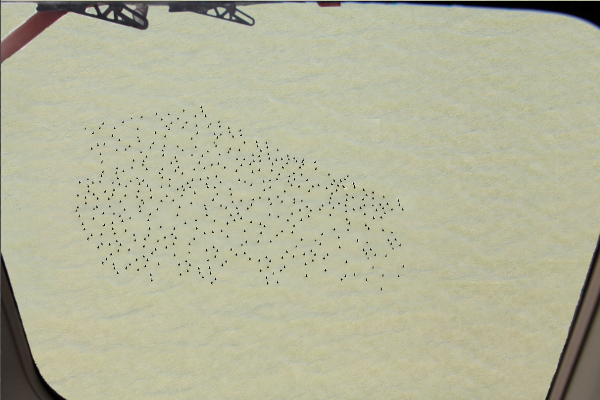


Q22. 363


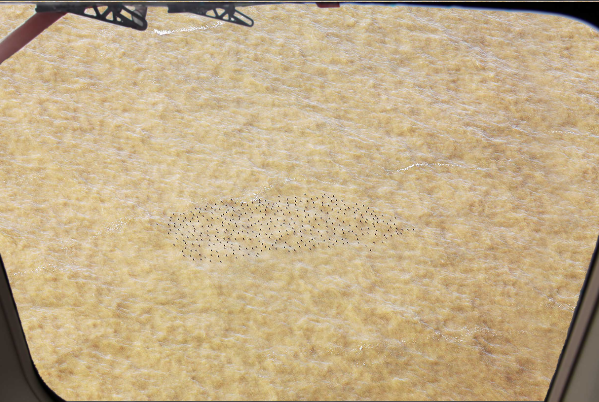

Supplement: Supplementary file 2 — Appendix S2 [file ECE3-12-e8733-s002.docx]
